# Advances in the pathogenesis of Rett syndrome using cell models

**DOI:** 10.1002/ame2.12236

**Published:** 2022-07-04

**Authors:** Sijia Lu, Yongchang Chen, Zhengbo Wang

**Affiliations:** ^1^ State Key Laboratory of Primate Biomedical Research, Institute of Primate Translational Medicine Kunming University of Science and Technology Kunming China; ^2^ Yunnan Key Laboratory of Primate Biomedical Research Kunming China

**Keywords:** cell models, MeCP2, pathogenesis, Rett syndrome

## Abstract

Rett syndrome (RTT) is a progressive neurodevelopmental disorder that occurs mainly in girls with a range of typical symptoms of autism spectrum disorders. MeCP2 protein loss‐of‐function in neural lineage cells is the main cause of RTT pathogenicity. As it is still hard to understand the mechanism of RTT on the basis of only clinical patients or animal models, cell models cultured *in vitro* play indispensable roles*.* Here we reviewed the research progress in the pathogenesis of RTT at the cellular level, summarized the preclinical‐research‐related applications, and prospected potential future development.

## Graphical abstract

Most Rett syndrome (RTT) patients are affected by the *MECP2* gene mutations. And the mutations of *MECP2* have wide effects on different cell types of nervous system, including neurons, astrocytes, oligodendrocytes and microglia, which always combined together to lead to abnormalities in the whole neural network.

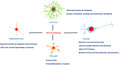

## 1 INTRODUCTION

Rett syndrome (RTT) is a neurodevelopmental disorder with certain symptoms of autism spectrum disorder (ASD).^1^, ^2^, ^3^, ^4^ Methyl‐CpG binding protein 2 (*MECP2*) was the first identified ASD‐causing gene. Mutations of the *MECP2* gene contributed most to the occurrence of RTT. Several other genes, such as *CDKL5* and *FOXG1*, are also identified as RTT‐causing genes that lead to atypical RTT.^5^, ^6^, ^7^, ^8^ Evidence shows that several neurodevelopmental disorders are related to dysfunction of MeCP2 protein expression.^9^ Therefore, the research progress of pathogenesis and treatment on RTT might be a wide reference to other ASD diseases. In this review, we focus mainly on the progress of *MECP2* mutant RTT.

Research on RTT has usually been based on patients or animal models. However, owing to ethical concerns, it is difficult to draw materials from clinical subjects, and animal models can simulate only partial phenotypes of clinical patients. Therefore, it is not enough to conduct in‐depth studies in these ways. It is necessary to use cellular models to study the comparatively systematic, complete mechanisms. Here we reviewed the important progress in the pathogenesis of RTT using cell models.

## 2 GENERAL INFORMATION ON RTT

**FIGURE 1:**
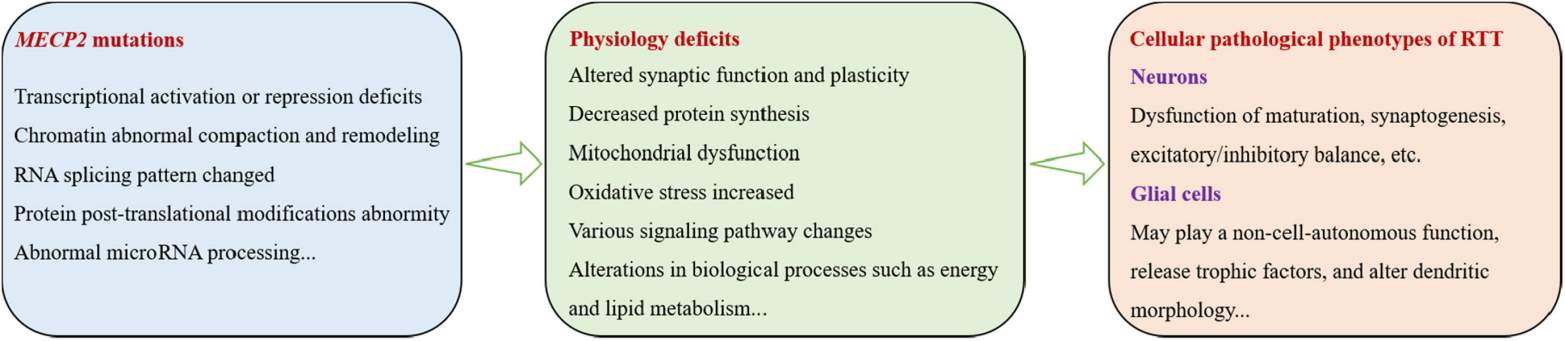
The effects of *MECP2* mutations on RTT. Multiple abnormal biological processes were regulated by *MECP2* mutations, which further lead to cellular physiological deficits in neurons and glial cells.

Mutations of the *MECP2* gene lead to MeCP2 protein loss of function in part or whole, which affects the methylation binding ability and regulatory function on gene expression, resulting in the phenotypes of typical RTT. Most patients with *MECP2* mutant RTT are female, with a prevalence of approximately 1/10 000–1/15 000.^1^ Most of the symptoms of patients with RTT occur in the central nervous system, including smaller brain volume and thinner cortical layer, which specifically present as smaller cell bodies, reduced spinous process density and complexity, and significantly lower overall neuronal maturity.^10^, ^11^, ^12^ These findings indicate that cellular‐level changes play an important role in RTT onset. We summarize the abnormal physiological processes, cell types, and pathological phenotypes affected by *MECP2* mutations in Figure 1. In addition, deficiency of this protein outside the nervous system can lead to lesions in the corresponding organs, such as cardiac, liver, and digestive tract, etc.,^13^ indicating that mutations of *MECP2* have complex functions throughout the body. Current research has mainly concerned damages in the nervous system.

As a transcriptional regulator, MeCP2 has a dual regulatory function, that is, transcriptional activation or inhibition. The severity of the RTT phenotype is related to the type of mutation.^14^, ^15^ Clinically, most mutation sites are located at the 2 functional domains of transcriptional repression domain (TRD) and methyl CpG binding domain (MBD),^16^ both resulting in severe RTT phenotypes. Previous studies have shown that the TRD and MBD domains are responsible for performing the primary functions of the protein, the NCoR/SMRT corepressor interaction domain (NID) exercises the function of the recruit's repressive complexes, and the AT‐hook domain (AT‐hook 1) assists in DNA bending and chromatin remodeling.^16^, ^17^, ^18^, ^19^, ^20^

## 3 A BRIEF OVERVIEW OF THE RESEARCH PROGRESS OF RTT ANIMAL MODELS

Rodent and nonhuman primate models are commonly used to study the disease progress and pathogenic mechanism of RTT. In 2001, Mecp2‐knockout mice were first reported,^21^, ^22^ which exhibit phenotypes resembling some of the symptoms of patients with RTT. In the conditional knockout mice, loss of Mecp2 in inhibitory neurons impaired the GABA signaling pathway, exhibiting autistic stereotypical behavior and severe phenotypes^23^; loss of Mecp2 in cholinergic circuits of basal forebrain and the striatum recapitulated some phenotypes.^24^ In addition, knockdown of Mecp2 in different brain regions of mice displayed different neuropathological phenotypes, suggesting a region‐specific effect.^16^ Mecp2‐deficient rat models generated in 2016 showed Rett‐like behavioral and motor deficits.^25^, ^26^ Subsequently, nonhuman primate models of RTT^27^ were constructed in 2017. Monkey models showed unique advantages in mimicking abnormal phenotypes of RTT in advanced cognitive, social behavior, and movement activity. They were also used to monitor brain development by neuroimaging.^27^ The abnormal development of white matter (WM) microstructure and network topological organization of monkey models may cause the RTT behavioral phenotypes.^28^ The above models have made a great contribution in tracking the disease progression and understanding the phenotypes of RTT. However, for the studies aimed to elucidate the mechanisms in living cells, or to carry out functional verification and pathogenesis exploration more conveniently and comprehensively, cell models are essential.

## 4 RESEARCH PROGRESS ON THE PATHOGENESIS OF RTT USING CELL MODELS

Most cells used in laboratory are usually derived from patients with RTT, animal models, or gene‐modified cells. The advent of drug studies based on induced pluripotent stem cells (iPSCs) is a milestone in nonclinical trials. Although only relatively few studies have used iPSCs derived from patients with RTT, there have been very important findings and research progress on pathogenesis and preclinical trials, as listed in Table 1.

**TABLE 1:** Main research progress of RTT pluripotent stem cells

Time	Researcher	Cell source	Mutation type	Main research
2010	Marchetto et al.^75^	Patient iPSCs	T158M, R306C, Q244X, 1155del32	Established an iPSC model derived from patients with RTT for the first time, and tested that IGF1 and gentamicin have a certain recovery effect on the number of glutamatergic synapses
2011	Kim et al.^30^	Female patients iPSCs	T158M, Q244X, E235fs, R306C, X487W	Defective neuronal maturation in *MECP2* mutants is associated with its non‐cell‐autonomous effects
2011	Cheung et al.^29^	Female patients iPSCs	Δ3–4 *MECP2* mutation，T158M,R306C	Δ3–4 mutation shows a random inactivation pattern of XCI, whereas point mutant cell lines showed a highly skewed pattern of XCI, and *MECP2* expression follows its XCI pattern
2011	Ananiev et al.^103^	Female patients iPSCs	T158M, V247X, R306C, R294X	Syngeneic controls isolated from iPSCs derived from patients with RTT for *in vitro* studies
2011	Amenduni et al.^104^	Male/female patients iPSCs	*CDKL5*: Q347X and T288I	The first use of *CDKL5* mutation in RTT cell modeling and neuron differentiation experiments; phenomenon of XCI
2013	Li et al.^81^	Gene editing ESCs	TALEN targets exon 3	*MECP2* deficiency leads to impaired AKT/mTOR pathway and mitochondrial function
2014	Williams et al.^49^	Female patients iPSCs	V247X, R294X and R306C	Coculture of normal neurons with *MECP2* mutant astrocytes and their conditioned medium exhibits neuronal deficits
2015	Djuric et al.^105^	Female patients iPSCs	*MECP2e1* mutations	*MECP2e1* mutations affect human neuron body size and electrophysiological properties
2015	Andoh‐Noda et al.^50^	Female patients iPSCs	Exon 4 (c.806delG)	TRD domain truncation of *MECP2* affects neuronal differentiation with a tendency to differentiate into astrocytes
2016	Tang et al.^35^	Male patients iPSCs	Q83X	*MECP2* mutation affects the expression of the downstream target gene *Kcc2*, resulting in GABAergic neuron dysfunction
2016	Chin et al.^106^	Patients iPSCs	R306C, 1155Δ32	Some pathological manifestations of RTT‐iPSC differentiated neurons can be alleviated by choline supplementation
2016	Delépine et al.^51^	Female patients iPSCs	R294X	EpoD can be used to improve the pathological changes of microtubule dynamics in *MECP2*‐deficient astrocytes, and enhancing microtubule stability may be a potential target for RTT therapy
2017	Yoo et al.^79^	Male patients iPSCs	Q83X	Restoring MeCP2 and L1 expression in RTT NPCs can normalize impaired neuritogenesis
2018	Landucci et al.^37^	Female patients iPSCs	T158M, R306C	RTT iPSC‐derived neuronal GABAergic circuits are upregulated; selective HDAC6 inhibitors ameliorate the reduction of acetylated α‐tubulin in RTT neurons
2018	Ohashi et al.^107^	Female patients iPSCs	705delG, X487W	The reduction in dendritic complexity of RTT neurons may be due to activation of the p53 pathway, or be associated with aging
2018	Mellios et al.^43^	Female patients iPSCs and male control	R106W, V247X	*MECP2* mutation upregulates the expression of mir‐199 and mir‐214, causing disturbance of ERK/MAPK and PKB/AKT signaling pathways, thereby affecting neurogenesis
2019	Souza et al.^108^	Male patients iPSCs	Q83X	The expression of TH‐related genes is altered in RTT
2019	Kim et al.^42^	Male patients iPSCs	Q83X, N126I	Proteomic analysis reveals the causes of dysregulated *LIN28* gene expression and delayed glial differentiation under *MECP2* mutation
2019	Sharma et al.^109^	Male patients iPSCs	Q83X	Exosomes extracted from normal cells, which carry the signaling information required to regulate the development of neural circuits, can alleviate the neural defects of RTT
2020	Varderidou‐Minasian et al.^41^	Patients iPSCs	*MECP2* exons 3–4 mutation	The findings provide a profile of proteomic changes in early neurodevelopmental stages (iPSCs to neuronal stem cells), suggesting that changes occur long before RTT syndrome symptoms become apparent
2020	Rodrigues et al.^40^	Patients iPSCs	Δ3–4 *MECP2* mutation，T158M,R306C and *MECP2e1* mutation	Translational ribosome affinity purification sequencing finds that the dynamic translationome in neural development is perturbed in RTT and proposes that alterations in ubiquitination may have therapeutic implications
2020	Gomes et al.^68^	Male/female patients iPSCs	R255X, Q83X	Premature development of the deep‐cortical layer of RTT forebrain organoids and functional deficits in RTT neurons; furthermore, the assembly of RTT dorsal and ventral organoids revealed impairments of interneuron's migration
2020	Xiang et al.^69^	Gene editing male ESCs	R133C, R270X, R306C	Adverse effects of *MECP2* mutant human cortical interneurons using 2D and 3D culture methods to rescue Rett‐like pathological phenotypes using BET inhibitor JQ1

*Note*: ERK/MAPK signaling pathways, extracellular signal‐regulated kinase signaling pathways; PKB/AKT signaling pathways, protein kinase B signaling pathways. The mutation type is mainly for the *MECP2* gene, and the mutations of other genes are specifically pointed out in the table.

Abbreviations: EpoD, epothilone D; ESCs, embryonic stem cells; iPSCs, induced pluripotent stem cells; RTT, Rett syndrome; TH, thyroid hormone; TRAP‐seq, translational ribosome affinity purification sequencing; XCI, X chromosome inactivation.

### 4.1 Research progress on RTT neurons

Neurons differentiated from iPSCs derived from patients with RTT show specific pathological phenotypes, that is, smaller neuron cell bodies, decreased synapses and spine density, and abnormal calcium signaling and electrophysiological function, reflecting important changes in the morphological structures and functions of RTT.^29^, ^30^ Disruption of the excitatory/inhibitory activity balance between synapses in different brain regions and circuits leads to an imbalance in microenvironmental homeostasis, which may lead to abnormal brain firing, resulting in epilepsy or other symptoms.^31^

Wild‐type iPSC‐derived neurons typically express high levels of synaptic adhesion molecule GluD1.^32^ MeCP2 deficiency caused changes in the action potential of glutamate neurons and a decrease in the number of synapses on glutamate neurons,^33^ implying that the fate of neural differentiation may shift to inhibitory neurons, which manifests itself in an increase in inhibitory synapses and a decrease in excitatory synaptic structures. This result may be caused by the downregulated expression of neuron‐specific membrane transporter K^+^/Cl^+^ cotransporter (KCC2) mediated by RE1‐silencing transcription factor (REST), a neuronal gene inhibitor in RTT, which is essential for maintaining excitatory balance in the brain.^34^, ^35^ Imbalance of excitatory/inhibitory circuits may be one of the important causes of RTT.^36^ In GABAergic neurons derived from the RTT‐iPSCs, GABAergic circuit disruption and decrease of acetylated α‐tubulin were also found.^37^

By analyzing and comparing the different stages of neural differentiation of RTT‐iPSCs, researchers revealed some changes and mechanisms at the molecular and cellular level. Transcriptome analysis showed that MeCP2 began to modulate before neural differentiation.^38^ During subsequent differentiation, forebrain neurons derived from several human RTT stem cell lines showed a reduction in the expression level of cAMP‐response element binding protein (CREB) and phosphorylated CREB, which could lead to functional defects in neurons.^39^ In RTT human iPSCs, neural progenitor cells, and cortical neurons, the expression of genes related to the dysregulation of mTOR signaling pathway and the ubiquitin pathway alters the structure of neurons, leading to defects in cell structure.^40^ Proteomic analysis revealed that both dendritic morphology and synaptogenesis‐related proteins were altered during RTT iPSC‐derived neuronal progenitors, that is, in early neuronal differentiation.^41^ Another study found that the *LIN28A* gene may participate in the regulation of neuronal differentiation in RTT‐iPSCs.^42^ Therefore, dysregulation of the expression of various genes and proteins during this early phase of neuronal differentiation may be an important reason for the progression of RTT.

In addition, MeCP2 also regulates microRNA (miRNA). The expression of miR‐199 and miR‐214 was found to be most significantly affected by the MeCP2 mutation. Restoration of miRNA expression in patients with RTT and MeCP2‐deficient neural stem/precursor cells can relieve the pathological phenotype of RTT neurons.^43^, ^44^

These results suggest that MeCP2 has a wide range of roles that may not only alter the substance transport process involved in organelles, but also adversely affect the formation and/or maintenance of neural processes by influencing the transcription. Although the effects of MeCP2 on normal brain development is not fully understood, there is no doubt that the mutation of MeCP2 disrupts the expression regulation of a large number of genes and the homeostasis of their microenvironment, which is an important premise of the RTT neuropathological phenotype.

### 4.2 Research progress on glial cells

**FIGURE 2:**
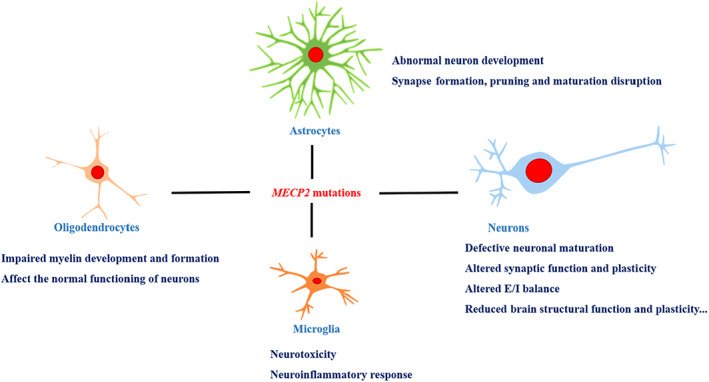
The effect of *MECP2* mutations on neural lineage cells. Mutations of *MECP2* have wide effects on different cell types of nervous system, including neurons, astrocytes, oligodendrocytes, and microglia, which combined lead to abnormalities throughout the neural network.

Previous studies have shown that RTT‐iPSCs mainly affect the differentiation and maturation of neurons. An increasing body of evidence demonstrates that glial cells also express MeCP2^45^, ^46^, ^47^ and MeCP2 deficiency in glial, like neurons, are integral components of the neuropathology of RTT^48^ (as shown in Figure 2). *Williams et al.* found that RTT astrocyte coculture with wild‐type neurons can impair normal neuron morphology and function.^49^ During neural differentiation, RTT neural precursor cells showed a tendency to differentiate into astrocytes.^50^ Microtubule dynamics stability was decreased in *Mecp2*‐deficient mouse astrocytes,^51^, ^52^ which may explain the impaired neurites observed in patients with RTT and in animal models.

The MeCP2 mutated microglia have a smaller soma body than wild type.^53^ Studies have shown that microglia affected by Mecp2 deficiency may be involved in the abnormal inflammatory response.^54^, ^55^ Enhanced oxidative stress and immune responses were found in both Mecp2‐null mice and their primary glial cells after lipopolysaccharide treatment.^56^ Likewise, persistent dysfunction of neurons or other glial cells also enhances the immune response of RTT microglia, which may further exacerbate the disease process.^57^ Higher levels of glutamate were detected in RTT microglia‐enriched conditioned medium, and addition of the medium to normal cultures also resulted in damage to dendrites and synapses in neurons.^58^ Mecp2 deficiency leads to overexpression of glutamine transporter (SNAT1), resulting in the production of large amounts of glutamine in mitochondria for metabolism and the formation of glutamate, which may be responsible for mitochondrial dysfunction and neurotoxicity.^59^ Other studies have shown that the involvement of miRNA in the regulation of the MECP2‐STAT3 axis or the modification of *MECP2* phosphorylation may also be the reason for the inflammatory response of microglia.^60^, ^61^

In mice, Mecp2 deficiency in the oligodendrocyte lineage also plays a unique role in the disease process of RTT.^62^ From isolated primary oligodendrocyte progenitor cells, the researchers found that the expression of MeCP2 increased in the maturation of oligodendrocyte differentiation process,^63^, ^64^ which confirmed that MeCP2 regulates myelin‐related genes, thereby affecting the process of oligodendrocytes participating in the formation of neuronal myelination.^65^ Therefore, it is believed that, under the influence of MeCP2 mutation, myelination‐related dysfunction in the central nervous system leads to RTT pathophysiology.

MeCP2‐mutant glial cells could affect the morphology and function of neurons, and affect the disease progression. However, the effects of MeCP2 on glial cells are currently less studied *in vivo* and *in vitro* than in neurons. Indeed, more than one cell type and function is regulated by MeCP2, but more evidence is needed to reveal its importance and role in the pathogenesis of RTT. Advances in the pathogenesis of RTT using cell models are summarized in Figure 2. Furthermore, the dynamics of connectivity and circuit neural networks during development and disease should be better examined with the development of new approaches^66^ (for a comparison of the advantages and disadvantages of cell culture methods, see Table 2), such as sparse coculture for connectivity (SparCon) assays^67^ or 3D organoid culture system.^68^, ^69^

**TABLE 2:** Comparison of 2D and 3D culture system for *in vitro* studies

Culture system	Advantages	Disadvantages	Research scope
2 Dimensional (2D) cells	Short‐term culture protocol; Easy to manipulation; Good repeatability.	Cell type is too single; Hard to simulate cell junctions and interaction; Difficult to mimic complex niches	They both can be used in the following research areas: Basic development; Cell physiology; Pathogenic mechanism; Potential therapeutic targets; Drug screening; Gene therapy; Cell therapy, etc.
3 Dimensional (3D) organoids	More similar to *in vivo* 3D stereoscopic environment; Simulate more complex physiological processes.	Long‐term culture requires the support of scaffolds, but their components are unclear; Difficult to directed differentiation; Less repeatability than 2D cells	

### 4.3 Research progress on treatment and functional recovery of RTT

At least 70 drugs have been reported in preclinical studies or clinical trials to ameliorate the symptoms of RTT, and some research has been systematically summarized.^70^, ^71^ Among the downstream signaling molecules regulated by MeCP2, several have been shown to have regulatory effects in RTT animal or cell models.

The point of treatment now is to improve the growth and development of neurons or restore their damaged neurites and synapses. Brain‐derived neurotrophic factor (BDNF) is a neurotrophic factor that plays an important role in neuronal survival and plasticity, and its expression is also regulated by MeCP2.^72^ The expression level was reduced in Mecp2‐deficient male mice, and when a certain level of expression was restored, the symptoms and lifespan of the diseased mice could be reversed,^73^ suggesting that treatments targeting the MeCP2‐BDNF axis in RTT could alleviate some symptoms and are potential therapeutic options for RTT. Protein tyrosine phosphatase‐1B (PTP1B) is a receptor for BDNF, and its pharmacological inhibition ameliorated the effects of *MECP2* disruption in RTT mice.^74^ When insulin‐like growth factor 1 (IGF‐1) and low concentrations of gentamicin were administered to RTT neurons, the morphology and function of damaged neurons could also be restored.^75^ Histone deacetylase 6 (HDAC6)‐selective inhibitors showed great application and therapeutic prospects to reverse the decreased microtubule acetylation in neurons of RTT.^76^, ^77^ Overexpression of L1 retrotransposon can partially restore neurite growth during RTT‐iPSC differentiation.^78^, ^79^ MeCP2's regulation of the multi‐subunit protein complex BLOC‐1 may also be a therapeutic target for synaptic dysfunction.^80^ The mutation impairs the neuronal AKT/mTOR pathway and mitochondrial function.^81^ Administration of the above pathways may improve the pathological phenotype of RTT neurons.

Restoring normal expression of *Mecp2* in the medial prefrontal cortex can improve behavioral deficits in mice.^82^ However, balancing the exogenous expression of MeCP2 to the physiological range remains a difficult problem to be solved, because the overexpression of MeCP2 leads to *MECP2* duplication syndrome.^71^ Another treatment is to reactivate the silent chromosomes that express the normal *MECP2* gene in the cells of female patients. In addition, noncoding RNAs participating in MeCP2‐related transcriptional regulation processes could also become therapeutic targets.^83^ Symptomatic treatment is one of the important strategies. RTT treatment in clinical and preclinical studies mainly include 2 approaches: one targets genetic and molecular pathology to repair the mutant *MECP2* or to regulate downstream molecules targeting related signaling pathways, such as growth factors (IGF‐1, BDNF), inhibitors (PTP1B, HDAC6), and other drugs for specific neuron types; the other alleviates the pathologic symptoms with physical stimulation interventions such as deep brain stimulation,^84^, ^85^ transcranial magnetic stimulation, etc. Nevertheless, none of these drugs have entered phase III/IV clinical trials. Emerging gene and cell therapy^86^, ^87^, ^88^, ^89^, ^90^ for repairing *MECP2* mutations *in vivo* or *ex vivo* are expected to provide a new treatment strategy after a series of evaluations from cells to animals, then preclinical trials. Moreover, MeCP2 mutations have widespread effects on the central nervous system, affecting all aspects of neurogenesis and biological processes. In‐depth analysis of the pathogenic mechanism of *MECP2* deficiency will facilitate the optimization and combination of the therapeutic approaches, such as the bias in early neural differentiation process, the exact mechanism of glial cells influencing neuronal structure and function, etc.

## 5 SUMMARY AND PROSPECTION

In‐depth study of RTT and MeCP2 has given us an understanding of MeCP2's multifunction: widely involved in the transcription regulation of genes, self‐translational modification in response to neuronal activity, and promotion of chromatin central aggregation,^91^, ^92^ etc., indicating its importance for individual neurodevelopment. Owing to the difficulties in pre‐onset data and sample collections from patients with RTT, the detailed mechanism of RTT in the early stages of postnatal development is still unknown. The emergence of iPSCs has brought great application prospects.^93^ To date, iPSCs provide a reusable, versatile, and consistent source from patients for *in vitro* studies of RTT. Although cell models are a powerful tool to investigate potential regulation mechanisms in RTT, neurodevelopment is a temporary and spatially related complex progress. Whether the results of *in vitro* and *in vivo* studies are highly consistent remains to be further demonstrated.

The understanding of RTT should not be limited to the severe consequences of its mutations; the function of MeCP2 in the nervous system and even in early ontogeny also needs to be understood. At present, 2D neural differentiation and 3D brain organoid culture technologies have been developed rapidly.^93^, ^94^, ^95^, ^96^, ^97^, ^98^ The combination of RTT‐iPSCs with the above technologies can be better applied to further research on RTT pathogenesis, drug screening, gene repair, and cell therapy. Taken together, the rational use of cells and other models^99^, ^100^, ^101^, ^102^ for research will help us to understand the pathogenic mechanism and develop new treatments for RTT in the future.

## AUTHOR CONTRIBUTIONS

Sijia Lu conceived and wrote the original draft of the manuscript. Zhengbo Wang and Yongchang Chen revised the manuscript. All authors critically read and contributed to the manuscript, and approved its final version.

## FUNDING INFORMATION

The National Natural Science Foundation of China (81930121, 31960120), the National Key Research and Development Program of China (2018YFA0107902, 2018YFA0801403) and the Major Basic Research Project of Science and Technology of Yunnan (202001BC070001, 202105AC160041)

## CONFLICT OF INTEREST

The authors declare that there is no conflict of interest regarding the publication of this article.
